# Baseline population health conditions ahead of a health system strengthening program in rural Madagascar

**DOI:** 10.1080/16549716.2017.1329961

**Published:** 2017-06-16

**Authors:** Ann C. Miller, Ranto H. Ramananjato, Andres Garchitorena, Victor R. Rabeza, Djordje Gikic, Amber Cripps, Laura Cordier, Hery-Tiana Rahaniraka Razanadrakato, Marius Randriamanambintsoa, Lara Hall, Megan Murray, Felicite Safara Razanavololo, Michael L. Rich, Matthew H. Bonds

**Affiliations:** ^a^ Department of Global Health and Social Medicine, Harvard Medical School, Boston, MA, USA; ^b^ PIVOT, Boston, MA, USA; ^c^ Institut National de la Statistique, Direction de la Demographie et de les Statistiques Sociales, Antananarivo, Madagascar; ^d^ Ministère de la Sante Publique, Region Vatovavy-Fitovinany, Manakara, Madagascar; ^e^ Department of Medicine, Stanford School of Medicine, Stanford, CA, USA

**Keywords:** Madagascar, health system strengthening, impact evaluation, under-5 mortality, maternal mortality, vaccination rates, baseline data, population survey, DHS, model district

## Abstract

**Background**: A model health district was initiated through a program of health system strengthening (HSS) in Ifanadiana District of southeastern Madagascar in 2014. We report population health indicators prior to initiation of the program.

**Methods**: A representative household survey based on the Demographic Health Survey was conducted using a two-stage cluster sampling design in two strata – the initial program catchment area and the future catchment area. Chi-squared and *t*-tests were used to compare data by stratum, using appropriate sampling weights. Madagascar data for comparison were taken from a 2013 national study.

**Results**: 1522 households were surveyed, representing 8310 individuals including 1635 women ages 15–49, 1685 men ages 15–59 and 1251 children under age 5. Maternal mortality rates in the district are 1044/100,000. 81% of women’s last childbirth deliveries were in the home; only 20% of deliveries were attended by a doctor or nurse/midwife (not different by stratum). 9.3% of women had their first birth by age 15, and 29.5% by age 18. Under-5 mortality rate is high: 145/1000 live births vs. 62/1000 nationally. 34.6% of children received all recommended vaccines by age 12 months (compared to 51.5% in Madagascar overall). In the 2 weeks prior to interview, approximately 28% of children under age 5 had acute respiratory infections of whom 34.7% were taken for care, and 14% of children had diarrhea of whom 56.6% were taken for care. Under-5 mortality, illness, care-seeking and vaccination rates were not significantly different between strata.

**Conclusions**: Indicators of population health and health care-seeking reveal low use of the formal health system, which could benefit from HSS. Data from this survey and from a longitudinal follow-up study will be used to target needed interventions, to assess change in the district and the impact of HSS on individual households and the population of the district.

## Background

Weak health systems foster illness, poverty, inequity and loss of human capital [1–3]. Disengagement, lack of resources (financial, human and supply chain), poorly integrated disease-specific (vertical) programs and lack of government support all contribute to the fragmentation of health systems. For example, a weak supply chain may mean that essential drugs and supplies are not available at a facility when patients arrive, which will neither help the individual patient, nor promote trust in the system. Likewise, lack of well-trained, supervised staff at health care facilities contributes to community disengagement from the health system. Patients may not return and essential services will not reach the community [4].

Most health system strengthening (HSS) interventions are designed to improve the six building blocks of an effective health system, which include: (1) effective and safe service delivery; (2) a well-performing and sufficient health workforce; (3) well-functioning information systems; (4) equitable access to medical care, products, vaccines and technologies and supplies; (5) health financing to ensure access and protect from catastrophic expenditures; and (6) effective leadership and governance [1]. The importance of robust impact evaluation measures to assess the effectiveness of these ambitious interventions has been increasingly clear in recent years [5], as have the challenges inherent in these kinds of studies, such as factoring in external actors that may be contributing in similar or synergistic ways to the population’s health [2], or accounting for rapid changes in socioeconomic progress regionally or nationally [3].

Lessons from clinical trials are relevant for the impact evaluation community – particularly that of the importance of transparency in identifying the outcomes targeted by interventions [6]. As with clinical trials, reporting bias in which published research focuses only on favorable or positive results can result in readers and policy-makers being informed only about programs that worked in some areas but not others, or that only appeared to be successful because of the use of an inferior comparison as the counterfactual [7,8]. To that end, a priori release of an evaluation strategy and baseline data is an important step towards transparency and increasing rigor in impact evaluation of complex interventions.

In 2014, a new health care organization, PIVOT, began operations in partnership with the Madagascar Ministry of Health (MoH) in the district of Ifanadiana, to create a model health district through a HSS program. In this paper, we report results from a cross-sectional baseline survey of the health conditions of the district population, discuss how the results will influence the program and describe the program’s intended evaluation structure.

## Methods

### Study population

Madagascar, an island nation off the east coast of the African continent, is one of the poorest countries in the world, with 81.7% of the population living on less than $1.90 USD per day in 2010 [9]. Ifanadiana District, in the southeastern region of Madagascar, consists of approximately 148,000 residents, gathered into 13 communes and 195 *fokontany* (the smallest administrative unit). Six of the communes are only accessible by foot or motorcycle. The district contains the eastern part of Ranomafana National Park, a montane rainforest and United Nations Educational, Scientific and Cultural Organization (UNESCO) World Heritage Site.

The HSS program based on the World Health Organization’s (WHO’s) six building blocks [1] supports the health system at three administrative levels: the district hospital; local health centers at the commune administrative level; and village community health workers at the *fokontany* level. Details on the activities of the program can be found in a forthcoming paper [10]. In brief, through partnership with the MoH, the program works at all levels of care (community health, health center and hospital) and includes the following interventions: infrastructure, staffing (quantity and capacity) and equipment (to strengthen service delivery and quality of care); strengthening procurement systems (to enable consistent drug and medical supplies); instituting an ambulance network (to improve physical access to emergency care); strengthening community health care (to assist in prevention, treatment and referral, improving access); implementing social programs (to institute needed services at village and commune level); removal of user fees (to remove financial barriers to access to care at all levels); and monitoring, evaluation and supervision (to strengthen all levels). Complementary to these interventions, specific clinical programs, including malnutrition, tuberculosis and maternal and child health, span all three levels of the health system.

The first phase of work from January 2014 through April 2016 covered 4 out of 13 communes – a population of about 65,000 people – with plans to progressively roll out services to the entire district. Although initial strengthening was not randomly allocated (for logistical reasons, the first sites chosen for the HSS initiative were those near Ranomafana National Park and along the main paved road in the district), this staggered roll-out of activities allows us to assess the impact of the HSS as it unfolds over time, in which some communes receive services first (‘initial catchment areas’) and others later (Rest of District, ‘RoD’). Before any of the program’s activities had begun (other than establishment of the formal partnership between PIVOT and the MoH), we conducted a baseline survey of the health and socio-economic conditions of the district.

### Survey data collection

**Figure 1.**
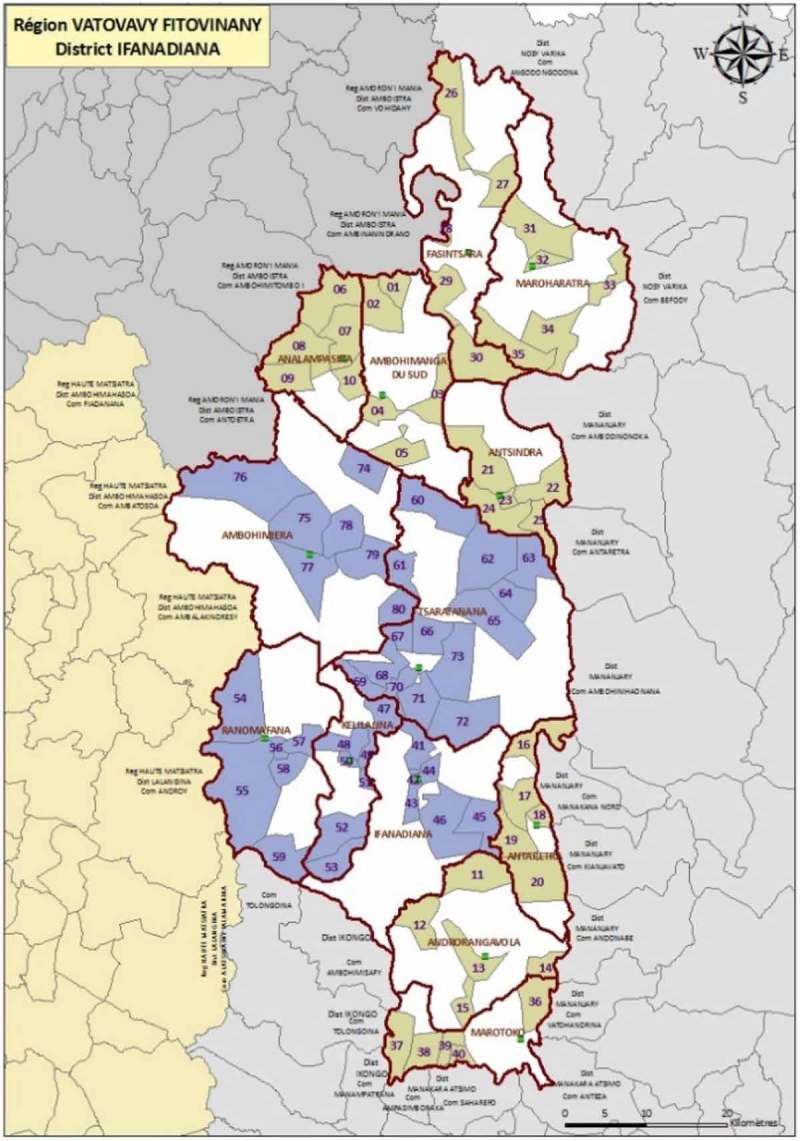
Sampling design used for the baseline survey in Ifanadiana District. Map shows the limits of the Ifanadiana District and its 13 communes. A total of 80 clusters were selected, half inside the initial PIVOT catchment area (blue) and half outside (tan), with probabilities proportional to the population size.

We conducted a representative survey of Ifanadiana District using tools and methods based on the Demographic and Health Surveys (DHS) [11], a project that conducts nationally representative household surveys in low- and middle-income countries (including Madagascar) approximately every five years to assess population, health and nutrition indicators. The survey was conducted by the Madagascar National Institute of Statistics (INSTAT), which also conducts the Madagascar DHS. Our sample consisted of 1600 households selected using a two-stage cluster sampling scheme involving 80 clusters and 2 strata (PIVOT’s initial area and RoD). Eighty clusters based on maps from the 2009 census were selected at random from the total available – 40 clusters within the initial catchment area and 40 from the RoD (Figure 1). The clusters were mapped and geographical information system (GIS) coordinates were collected for each cluster. Twenty households per cluster were randomly selected for inclusion. Five teams of five (four data collectors and one field supervisor per team) conducted face-to-face surveys with participating households in April and May of 2014. Eligibility criteria for interview were based on DHS standard criteria and included individuals of reproductive age (defined as the age ranges of 15–49 for women, 15–59 for men) who were de facto residents of the household (usual members or had spent the night prior in the household). Sensitization of the community prior to field work was performed through meetings with local mayors, government and MoH representatives and village leaders.

### Indicators

Our indicators of interest for health and economic well-being were defined a priori and are presented in Box 1. Core indicators were chosen to represent key access- and health- related factors that a strengthened health system is expected to influence, and were based on those from the Doris Duke Charitable Foundation’s African Health Initiative Population Health Implementation and Training (PHIT) partnership [12–14]. For example, strengthening community health services and health worker training and capacitation have been shown to reduce neonatal and infant mortality [15], and a 2014 study found that elimination of user fees increased care coverage, with accompanying substantial reductions in estimated neonatal and child mortality [16]. Our indicators for care access and utilization outcomes include vaccination rates (DTP [diphtheria-tetanus-pertussis] – at least one dose and all three recommended doses, measles, and full immunization coverage by 24 months), care-seeking for children’s illnesses (defined as treatment sought from trained health professional or clinic for diarrhea, acute respiratory illness, and fever), antenatal care and delivery with a trained health professional. Our indicators for population health impact include under-five mortality, under-five children with diarrheal, febrile or acute respiratory infection (ARI – defined as cough with shallow, rapid breathing), illness in the last two weeks and maternal mortality.

### Data collection and management

We collected the following data: basic household composition and living conditions, including gender, ages and relationships among household members; structure and physical condition of the house; type and condition of latrine or toilet; electricity; and access to water, among others. Child health characteristics included date of birth, nutrition, anthropometry (height and weight), vaccination status, health insurance, diarrheal or febrile illness within two weeks prior to interview, access to selected health services, treatment for malaria, or presence of an illness requiring a visit to a health center in the last three months  prior to the interview. Adult health characteristics included measures of febrile and diarrheal illness, injuries, respiratory illness, other chronic and acute conditions, anthropometry, health care utilization and reproductive history. We also collected information on adult and under-five mortality [17]. Economic data collected included 30-day and 12-month income and expenditure data, employment data, household goods, land and livestock ownership, and time lost to work or education because of illness or injury.

Our questionnaires were a subset of those used in Madagascar for the DHS [11], with added questions on child development from the Malagasy version of Multiple Indicator Cluster Survey-version 4 (MICS) [18] and questions on adult health from Rwanda’s Questionnaire de Bien-Etre [19]. Economic status was assessed using tools adapted from the World Bank’s Living Standard Measurement Survey (EICV) [20]. We successfully conducted a pilot of tools and methods in February 2014 in 80 rural households in a different district. All French and Malagasy questionnaires, data collection and analysis methods were standardized and had been reviewed and approved the year prior to the study by the Madagascar National Ethics Committee and re-reviewed through internal processes at INSTAT. The study was also reviewed by the Harvard Medical School Institutional Review Board (IRB). All individual identifiers were removed prior to database delivery to the Investigators. The ongoing longitudinal component of the study was re-reviewed and approved by Harvard Medical School IRB and the Madagascar National Ethics Committee.

All eligible women and men who were in the households sampled were interviewed. All residents, including children, were weighed and had their height measured (or, in the case of infants, length). No biologic samples were taken.

All health indicators were defined using the standard techniques of the DHS or MICS and are presented in Box 1. Under-five mortality was estimated using the synthetic life-table method in which probabilities of death are calculated for small age segments, then combined into overall mortality rates [17]. Under-five mortality was defined as deaths per 1000 live-born children ages 0–59 months and 29 days. Maternal mortality was estimated using the sisterhood method [17]. In brief, women were asked about the deaths of their sisters who were older than 15 years at their demises. Deaths during delivery or within two months of the end of a pregnancy were considered to be deaths due to maternal causes. Vaccination rates were defined as the proportion of children aged 12–23 months who received all recommended vaccines prior to their first birthdays. Incidence of childhood illness was defined as the proportion of children under five years of age who were ill with a specific condition in the two weeks prior to the survey.

**Box 1.** Health indicators, Ifanadiana District, Madagascar, 2014.

Category	Indicator	Description
Impact: Mortality	Under-5 mortality	Number of children who died before 5th birthday per 1000 live births
Impact: Mortality	Maternal mortality	Women ages 15–49 who died within 12 months of the end of a pregnancy per women with a reported pregnancy
Impact: Nutrition	Stunting prevalence	Number of children under age 5 who are < –2 SD of median height-for-age of World Health Organization (WHO) standard
Outcome: Immunization	Full immunization coverage	Number of children ages 12–23 months who received all recommended vaccines before first birthday
Outcome: Immunization	Measles vaccine coverage	Number of children ages 12–23 months who received measles vaccines before first birthday
Outcome/Impact: Child illness	Diarrheal incidence	Number of children under age 5 with diarrheal illness in last 2 weeks
Outcome: Child illness	Care-seeking for diarrheal illness	Number of children under age 5 with diarrheal illness in last 2 weeks for whom advice or treatment was sought from health facility or provider
Outcome/Impact: Child illness	Fever incidence	Number of children under age 5 with fever in last 2 weeks
Outcome: Child illness	Care-seeking for fever	Number of children under age 5 with fever in last 2 weeks for whom advice or treatment was sought from health facility or provider
Outcome/Impact: Child illness	Acute Respiratory Infection (ARI) incidence	Number of children under age 5 with cough in last 2 weeks
Outcome: Child illness	Care-seeking for ARI	Number of children under age 5 with cough in last 2 weeks for whom advice or treatment was sought from health facility or provider
Outcome: Malaria prevention	Bed net use	Number of children under age 5 who slept under bed net last night
Outcome: Reproductive health	Antenatal care coverage	Number of women 15–49 with a live birth in last 2 years who were attended at least 1 time by a skilled provider for antenatal care for the last birth
Outcome: Reproductive health	Skilled attendant at delivery	Number of women 15–49 with a live birth in last 2 years who were attended by a skilled provider during the last birth
Outcome: Economic	Wealth quintile	Value of household wealth index closest to but less than 20%, 40%, 60% and 80% of the cumulative distribution

Wealth indices were calculated as in the DHS method, using principal components analysis [17]. Factors that comprised the wealth index included access to electricity, water and toilets, material of roofing for houses, number of residents per bedroom and type of cooking fuel. Cutoff points for wealth quintiles were determined as the values of the wealth index closest to but less than 20%, 40%, 60%, and 80% of the cumulative distribution of the household wealth index.

### Data analysis

Data were entered into CSPro and analyzed using SPSS (SPSS Inc., Armonk, NY), Stata 13.1 (StataCorp, College Park, TX) and SAS® 9.3 (SAS Institute, Inc., Cary, NC) to calculate under-five mortality 95% confidence intervals. Sampling weights were calculated for the household, women’s and men’s surveys. To compare data between strata, we conducted chi-squared and *t*-tests for categorical and continuous data, respectively. All analyses, including the estimation of means, standard errors and tests, used the survey procedures available in Stata, applying appropriate sampling weights and using Taylor linearized variance estimation.

To compare our data to national estimates, we used reported estimates from the 2012–2013 ENSOMD (Enquete Nationale sur le Suivi des Objectifs du Millenaire pour le Developpement a Madagascar) survey for comparison [21–23]. ENSOMD, a nationally representative health and economic survey of 75,931 individuals in 16,920 households, was conducted in Madagascar in 2012 and 2013 to assess progress toward meeting the Millennium Development Goals. A report on progress toward each of seven goals is publicly available (http://www.mg.undp.org/content/madagascar/fr/home/library/mdg/publication_1.html). Raw data from this survey were not available, so statistical analyses of differences between district- and national-level estimates are not provided.

## Results

Data were collected from 1522 of 1600 households with a response rate of 95.5%. In these 1522 households surveyed, 1774 women and 1863 men were identified as eligible for individual investigation. 49.9% of the population of Ifanadiana is under age 15, with 17.0% under age five. These distributions were approximately the same for the initial area and RoD and similar to Madagascar overall with 47% under age 15 nationally and 16% under age five. Tables 1 and 2 show estimates of socio-economic and health indicators for Ifanadiana District overall compared to the initial catchment area vs. the RoD and to national estimates from ENSOMD for reference.

**Table 1.** Socio-economic indicators in the population of Ifanadiana District, 2014.

Socio-economic indicators	District estimate	Madagascar	Initial catchment	Rest of District (RoD)	*p*-value (initial vs. RoD)
Household characteristics					
Household size (N)	5.2	4.6	5.3	5.6	N/A
Female-headed household (%)	17.8	22.0	20.0	15.6	0.24
Primary occupation of household head is agriculture (%)	84.8	63.1	75.9	93.4	< 0.001
Access to improved sanitation (%)	2.9	N/A	5.2	0.7	< 0.001
Access to safe drinking water (%)	14.9	N/A	24.9	4.9	< 0.001
Households with at least 1 bed net (%)	94.8	64.5	93.7	96.0	0.08
Proportion of households in lowest wealth quintile	N/A	N/A	25.0	14.8	< 0.01

**Table 2.** Baseline health indicators in the population of Ifanadiana District, Madagascar, 2014; overall and with a comparison by catchment area.

Health indicators	District estimate	Madagascar*	Initial catchment	Rest of District (RoD)	*p*-value (Initial vs. RoD)
**Child health**					
Under-5 mortality (5 year estimate)	145/1000 live births	62/1000	134/1000	155/1000	
Children 11–23 months with all vaccinations (%)	34.6	51.1	39.9	29.5	0.27
Children < 5 with ARI in last 2 weeks (%)	27.8	10.7	28.1	27.6	0.89
Children with ARI taken for care (%)	34.7	40.5	34.8	34.6	0.68
Children < 5 with diarrhea in last 2 weeks (%)	14.3	11.3	14.2	14.8	0.70
Children with diarrhea taken for care (%)	56.6	44.5	55.1	58.1	0.15
Children < 5 with fever in last 2 weeks (%)	33.5	13.8	27.6	38.7	0.02
Children with fever taken for care (%)	42.5	47.7	49.1	38.6	0.013
Children < 5 moderately to severely stunted (%)	51.3	47.3	43.9	56.1	0.29
**Adult health (age 15+)**					
Male mortality (2007–2014) ages 15–49	5.5/1000	4.9/1000	**	**	
Female mortality (2007–2014) ages 15–49	7.5/1000	4.1/1000	**	**	
Maternal mortality (2007–2014)	1044/100,000 births	478/100,000	**	**	
Illness in last month (%)	46.3	N/A	47.2	46.3	0.8
Illness prevented work in last month (%)	71.7	N/A	66.4	77	< 0.01
Mother got at least 1 ANC visit (%)	78.9	82.1	79.2	78.7	0.68
Mother got 4 ANC visits (%)	36.4	57.3	42.0	31.4	0.07
Mother delivered last baby in health care facility (%)	18.4	43.0	15.4	21.7	0.33
Mother had trained help delivering last baby (%)	19.9	59.0	17.3	23.0	0.36
Mother had postnatal care within 2 days (%)	57.1	62.2	51.6	62.0	0.09

*Data from Madagascar from the 2013 ENSOMD studies, available at (http://www.mg.undp.org/content/madagascar/fr/home/library/mdg/publication_1.html **numbers too small for reliable comparisons

### Household characteristics

The mean household size in Ifanadiana is 5.2 residents, larger than the 4.6 residents for Madagascar’s rural population overall. 14.9% of the district gets drinking water from an improved or protected source and 2.4% of the population has access to an improved toilet facility (either a flush toilet or latrine with walls, roof and a washable slab). The initial catchment area has significantly higher access to improved drinking water, toilets or improved latrines and electricity, and a higher proportion of female-headed households than the RoD. Mean household size and proportion of households in the lowest wealth quintile did not differ significantly between the two strata. Overall, 94.8% of households in Ifanadiana District have at least one mosquito bed net. This was higher than the national estimate of 64.5%. There was no difference in bed net ownership between the initial catchment area and RoD.

Agricultural assets were not significantly differently distributed between the initial catchment area and RoD. Eighty-five percent of households overall have arable land for farming; 82.3% of initial catchment households vs. 88% of RoD households have arable land. 76.1% of the population has farm animals of some kind – 73.1% of initial catchment households vs. 79.2% of households in the RoD. Statistically significant differences existed in wealth category between the initial catchment and RoD strata, with 76% of the richest quintile being in the initial catchment and 63% of the poorest quintile being in the RoD.

### Child health

Under-five mortality in Ifanadiana District was estimated at 145 deaths per 1000 live births (using five-year estimates). This estimate is substantially higher than the national estimate from the ENSOMD of 62 deaths per 1000 live births [22]. This estimate was not statistically significantly different by strata; estimates were 134/1000 (95% CI 102, 166) in the initial catchment area and 155/1000 (95% CI 108, 202) in the RoD.

Diarrheal illness, malaria and ARI are the three leading causes of child mortality in Madagascar [24]. In the two weeks prior to the interview, approximately 14% of children under age five were reported to have had diarrheal illness, 34% were reported to have had fever and 27.8% ARI. These are also higher than national estimates from 2013; 11.3% of children in Madagascar were estimated to have diarrhea, 13.8% to have fever, and 10.7% to have ARI. Statistically significant differences in the percentage of children with fever existed between the initial catchment area and RoD; 38.7% of children in the RoD had fever vs. 27.6% of those in the initial catchment area (*p* < 0.05). No differences between the initial catchment area and RoD existed for diarrhea or ARI. Of those with illnesses in the two weeks prior to the survey, there was no difference in the proportion of children whose parents sought care for their illnesses.

Chronic undernutrition or ‘stunting’ (height-for-age [HFA] more than two standard deviations below a z-score normed for a well-nourished population of children of the same age and gender) is a problem in Ifanadiana District with about half of the children ages 6–59 months (51.3%) classified as stunted and 21.1% as severely stunted (HFA z-score of < −3 standard deviations). These are somewhat higher than national estimates of 47.3% stunted and 18.1% severely stunted. Although there was no statistically significant difference in overall stunting between the initial catchment area and RoD, children in the initial catchment area were less likely to have severe stunting than children in the RoD (17.0% initial catchment area vs. 24.6% RoD, *p* = 0.03).

Overall, 29.7% of children between 12 and 23 months in Ifanadiana District had received all of their required vaccinations as of 12 months (one BCG, three DTP, measles vaccine and three polio1), compared with 51.1% nationally [22,p.22]. No differences were seen in proportions of children receiving measles vaccine; however, a significantly higher percentage of children in the initial catchment area received a third DTP vaccine (67.4% in the initial catchment area vs. 48.1% outside, *p* = 0.04). The initial catchment area had higher estimates of third dose of DTP vaccines than the national estimates of 63.9% [17,p.22].

### Adult health

Among the 4104 adults surveyed, 46.7% reported having had an illness or injury in the 4 weeks prior to the survey, and 19.9% reported dental problems; these prevalences did not vary between the study groups. However, 66.2% of adults in the initial catchment area reported having missed school or work because of the illness or injury compared to 77.4% of those in the outside area (*p* = 0.02). Men were significantly more likely to report injuries than women (8.3% male vs. 2.6% female, *p* < 0.01).

### Reproductive health

The maternal mortality rate estimated for the Ifanadiana District was 1044 per 100,000 live births in our study, which is more than twice as high as the national estimate of 478 per 100,000. Of women who had given birth in the last 5 years, most (80.6%) gave birth at home. 18.3% gave birth to their most recent child in a health facility. This was not significantly different between the initial catchment area (21.7%) and RoD (15.4%, *p* = 0.33). 19.9% had professionally trained assistance (from a doctor, nurse or professionally trained midwife) in delivering their last baby. Traditional birth attendants assisted at 71.1% of the last births in the last five years – this also was not statistically significantly different by stratum (*p* = 0.18). These numbers are strikingly different than national estimates, with 57.0% of Malagasy women nationally giving birth at home and 41% assisted by traditional birth attendants [18]. Overall, 78.9% of women in Ifanadiana ages 15–49 had at least one pre-natal visit during their most recent pregnancy in the last 5 years (vs. national Madagascar estimates of 82.1%). However, only 36.4% of women received the recommended four ANC visits during that time. National estimates show that 57.3% of women in Madagascar receive at least four ANC visits. Neither of these estimates was statistically significantly different by stratum (Table 2). Fifty-seven percent of women received some kind of post-natal care after their last birth, most from traditional healers, compared with 62.2% of women nationally.

## Discussion

Through the execution of a standardized, well-established district-wide population survey conducted by experienced Malagasy professionals, we were able to document the health and economic status of the District of Ifanadiana at baseline of a comprehensive HSS program. Our data confirm that conditions in Ifanadiana fall below national estimates, and the collaborative efforts of the MoH, PIVOT and other partners to improve these conditions are needed. We publish here baseline data to highlight the needs of the district and to provide a benchmark for accountability to the population of Ifanadiana.

A major finding of our baseline survey is the high rates of maternal and under-five mortality in the district, which are more than double the national estimates for Madagascar and are nearly twice the estimates for sub-Saharan Africa in 2014 as a whole (maternal mortality 560/100,000 live births, under-five mortality 86/1000 live births [25]). Improving the very low vaccination rates in Ifanadiana can help to reduce child mortality from preventable causes, and bring both women and children into the strengthened health care system. Furthermore, 81.0% of pregnant women in the study delivered their babies at home, and most women (71.1%) are aided during delivery by traditional birth attendants rather than trained providers. This is much higher than the national average in Madagascar [23] and may contribute to the very high maternal mortality risk. A 2006 *Lancet* series on maternal mortality highlights the importance of intra-partum care: ‘most maternal deaths occur during labour, delivery or the first 24 hours postpartum and most complications cannot be predicted or prevented’ [26,p.1291]. Delivery in a well-staffed and capacitated health center would facilitate response to certain deadly complications. Additionally, home delivery even in wealthy countries has been linked to higher rates of neonatal mortality [27,28]. These data support the importance of the strengthened services PIVOT and MoH have undertaken since 2014, including the ambulance network, staffing, equipment, infrastructure improvements and supply chain management. Encouraging women into well-functioning health care facilities for delivery will also require acknowledgement of the importance of traditional attendants in the community and birthing process with some contingency plans for their linkage with the formal health system.

In our study, only one-third of children aged over 23 months were fully vaccinated on schedule. Differences in vaccination rates by study area became apparent for the third vaccinations in a series. 71.1% of the working and school-age population had lost some time at work or school in the last month because of illness or injury and differences in time lost because of illness between the catchment areas were apparent as well. Given that many of the villages in the RoD are farther from the roads and clinics than the initial catchment area, distance probably plays a role in both the differential vaccination rates and work/school time loss. Distance to services [29], wealth [30,31] and positive views toward health facilities are associated with full vaccination in multiple studies; other factors in studies associated with vaccination status include mother’s health and reproductive care [32,33]. Nevertheless, predictors of full vaccination vary across different contexts [33]. This highlights the importance of understanding local context to try to address these factors. High rates of malnutrition in children and adults (adult data not shown) also likely have an impact on both acquisition of illness and mortality. PIVOT and MoH’s program (started in October 2014) to eliminate out-of-pocket payments and ensure that essential medicines are in-stock may help facilitate a positive view of the health system and result in an increase in vaccination coverage over time. A malnutrition program was initiated in the fall of 2015 in health centers of the initial catchment area to address the high rates of severe acute malnutrition, as the baseline survey estimated. Future waves of the survey will help to assess the impact of these programs on the study participants and the district as a whole.

Because the HSS effort is aimed to progressively scale up services across the entire district, we do not face the common ethical concern that researchers are withholding services from some group of people for the duration of the study, as can happen in randomized controlled trials. However, this adds complexity to the evaluation of impact and necessitates clear knowledge of the evolution of health care access and health indicators over time in the different parts of the district. We have accordingly begun a longitudinal survey, enrolling the same households from the baseline study, with annual data collection to determine whether anticipated services are reaching the people we expect, and to assess the effect of the services on the people receiving them for varying periods of time. Data collection for Wave Two of the study was completed in  October 2016. One limitation to our study design is that although selection of villages for our baseline survey was random, selection of the initial program catchment area was not. Though the majority of indicators were not statistically significantly better for the initial catchment area than the RoD, it is not surprising that there are some statistically significantly different indicators attributable to the economic advantages of proximity to the park and main road. People outside of the initial catchment area are poorer and, in some cases, have worse population health indicators. We anticipate that our longitudinal study design, in combination with difference-in-differences calculations and decomposition analyses, will allow for rigorous estimates of the impact of services on the health of the population.

## Conclusion

The ultimate goal of this HSS initiative is to establish an evidence-based model health district for the country of Madagascar. A baseline survey led by the National Institute of Statistics according to international standards, was successfully conducted prior to initiation of the activities in Ifanadiana, Madagascar, documenting the population’s need and providing reliable estimates of health and socio-economic indicators at the district level. Ifanadiana’s population is, on the whole, poorer and less healthy than the estimates for Madagascar as a whole, a country with some of the poorest economic and health indicators in the world. This is the first step of a longitudinal study that will include follow-up of the population, which will help to inform future activities for the MoH, PIVOT and partners in Ifanadiana district, and will allow for a comprehensive impact evaluation.

## Availability of data and materials

The data that support the findings of this study are available from PIVOT and/or INSTAT but restrictions apply to the availability of these data, which were used under license for the current study and so are not publicly available. Data are however available from the authors upon reasonable request and with the permission of PIVOT and INSTAT.

## Paper context

Complex health system strengthening (HSS) programs integrate many levels of a system, and are hoped to have positive impacts on population health. Impacts, however, are difficult to measure and often baseline data for comparison are lacking. We report health indicators from a baseline population survey and an evaluation plan for a HSS program undertaken in a model district in southeastern Madagascar, and compare initial intervention areas with the rest of the district and all of Madagascar.
